# Energy Efficient Neural Stimulation: Coupling Circuit Design and Membrane Biophysics

**DOI:** 10.1371/journal.pone.0051901

**Published:** 2012-12-13

**Authors:** Thomas J. Foutz, D. Michael Ackermann Jr., Kevin L. Kilgore, Cameron C. McIntyre

**Affiliations:** 1 Department of Biomedical Engineering, Case Western Reserve University, Cleveland, Ohio, United States of America; 2 Department of Biomedical Engineering, Cleveland Clinic Foundation, Cleveland, Ohio, United States of America; 3 MetroHealth Medical Center, Cleveland, Ohio, United States of America; 4 Biodesign, Stanford University, Palo Alto, California, United States of America; 5 Louis Stokes Cleveland Veterans Affairs Medical Center, Cleveland, Ohio, United States of America; Hospital Nacional de Parapléjicos, Spain

## Abstract

The delivery of therapeutic levels of electrical current to neural tissue is a well-established treatment for numerous indications such as Parkinson’s disease and chronic pain. While the neuromodulation medical device industry has experienced steady clinical growth over the last two decades, much of the core technology underlying implanted pulse generators remain unchanged. In this study we propose some new methods for achieving increased energy-efficiency during neural stimulation. The first method exploits the biophysical features of excitable tissue through the use of a centered-triangular stimulation waveform. Neural activation with this waveform is achieved with a statistically significant reduction in energy compared to traditional rectangular waveforms. The second method demonstrates energy savings that could be achieved by advanced circuitry design. We show that the traditional practice of using a fixed compliance voltage for constant-current stimulation results in substantial energy loss. A portion of this energy can be recuperated by adjusting the compliance voltage to real-time requirements. Lastly, we demonstrate the potential impact of axon fiber diameter on defining the energy-optimal pulse-width for stimulation. When designing implantable pulse generators for energy efficiency, we propose that the future combination of a variable compliance system, a centered-triangular stimulus waveform, and an axon diameter specific stimulation pulse-width has great potential to reduce energy consumption and prolong battery life in neuromodulation devices.

## Introduction

The delivery of therapeutic levels of electrical current to excitable tissue is a well-established treatment for numerous diseases and disorders. Neuromodulation devices have been implanted in hundreds of thousands of patients for numerous FDA approved applications that include deep brain stimulation (DBS) [1], spinal cord stimulation (SCS) [2] and cochlear stimulation [3]. Clinical neuromodulation technologies typically rely on an implanted pulse generator (IPG) to deliver electrical stimuli. The fundamental goal of stimulation is to generate action potentials in axons near the implanted electrode that modulate the release of neurotransmitters in specific parts of the nervous system. However, the basic stimulation hardware and stimulus waveform used in clinical practice have remained relatively unchanged for decades. We propose that there exist numerous opportunities to improve IPG function, particularly to increase the energy efficiency of stimulation. This study presents a range of methods that decrease power consumption by exploiting the biophysical features of stimulated tissue, and minimizing the energy consumption of the IPG electronic circuitry.

Clinical neuromodulation devices are typically powered using a hermetically sealed medical grade energy cell (battery) that is located within the IPG package. The cell’s energy capacity and lifetime is dictated by its size (volume) and chemistry. The cell’s lifetime (or recharge interval for rechargeable cells) is dictated by the energy capacity and energy depletion rate across each of the functional circuit elements. The stimulation circuitry typically consumes the largest share of the energy; hence, its design and implementation represent compelling targets for optimizing IPG energy consumption.

**Figure 1:**
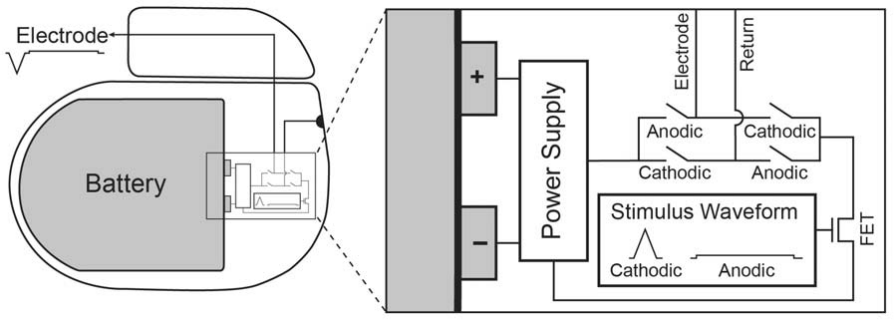
Implantable pulse generator. The constant-current source (FET) draws current from the battery-powered supply. A waveform generator provides the shape of the cathodic and anodic waveform, driving a transistor output stage. Transistor switches are used to select the waveform polarity (cathodic and anodic).

The power consumed during constant-current electrical stimulation is largely dictated by two fundamental elements: the current source and the electrode/tissue load. During stimulation, a current is applied across the tissue. The current source is typically implemented with transistor technologies to deliver a constant current over the stimulus pulse, regardless of the voltage generated across the electrodes. Traditional constant-current stimulators rely on a fixed compliance voltage (see Figure 1) that provides the driving potential to deliver current across the tissue load. This compliance voltage is application dependent and is generally fixed to a value larger than absolutely necessary for the IPG current output specifications (e.g. stimulation with 10–25 V) [4]. This safety margin enables the device to operate normally even under extreme operating conditions of large load impedances or large stimulation current thresholds in a given patient. However, under normal operating conditions, this fixed high compliance voltage is excessive for stimulating the necessary neural tissue. In turn, the stimulator operates inefficiently due to the large voltage drop across the current source.

When considering the neural tissue itself in the energy calculations, energy savings are also possible by optimizing the stimulus waveform shape and pulse duration used to generate action potentials with electrical stimulation [5], [6]. We recently reported on a computational model of neural stimulation where non-rectangular waveforms could achieve an energy savings of ∼10% compared to an optimal rectangular pulse [7]. We found that the most energy-efficient waveform shape was a centered-triangular pulse (ramp-up phase followed by a symmetric ramp-down phase); however, *in vivo* verification of these theoretical savings has not been previously demonstrated.

In this study we independently investigated multiple methodologies for maximizing the energy efficiency of a neural stimulator by exploiting both the biophysical features of stimulated tissue and the electronic circuit design of the IPG. We utilized measurements acquired in an *in vivo* rat preparation and in computer simulation to quantify the energy consumed by the current source and tissue/electrode load. We investigated the energy content of stimulation with different waveform shapes and pulse-widths, as well as analyzing the impact of minimizing the compliance voltage on IPG energy consumption. We found that the use of an adjustable compliance voltage in the pulse generator circuitry, and a biophysically optimized triangular stimulus waveform and pulse-width, can independently reduce energy consumption in neurostimulation applications.

## Methods

### Ethics Statement

All experiments involving animals were approved by the Case Western Reserve University institutional animal care and use committee (protocol #2008-0011), and built upon an established rat sciatic nerve preparation [8].

### Overview

Two series of experiments were performed to compare the energy required for traditional neural stimulation (rectangular waveform, fixed compliance voltage) versus (1) an energy-efficient centered-triangular waveform, or (2) an adjustable compliance voltage. Experiments were performed in adult Sprague-Dawley rats, with unilateral peripheral nerve stimulation. Both experiments spanned a large range of stimulation pulse-widths (PWs) to investigate its impact on energy consumption. Finally, computer simulations of peripheral nerve stimulation were performed to evaluate the role of axon fiber diameter on the energy-optimal pulse-width for activation.

### Electrode Placement and Stimulation Procedure

**Figure 2:**
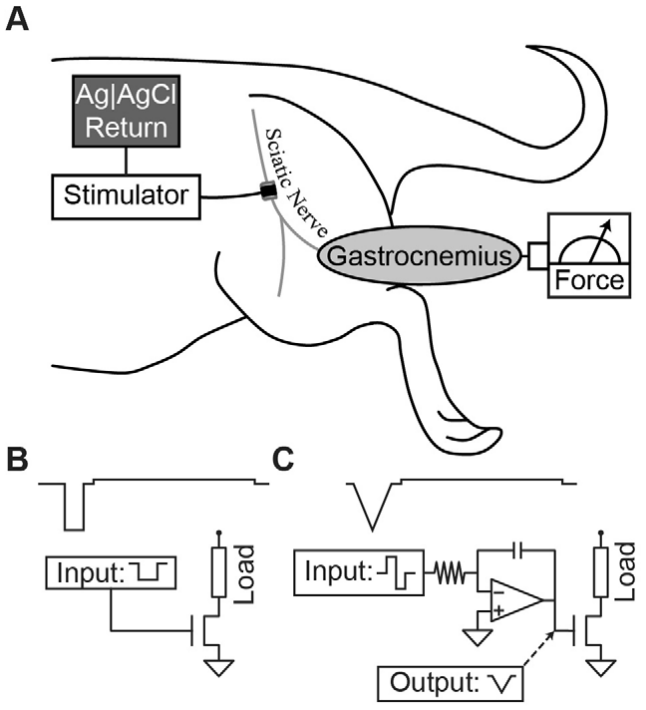
Experimental setup. (A) Stimulation of the sciatic nerve induces contraction of the gastrocnemius muscle. Force is quantified by a force transducer. (B) Rectangular stimulus waveform. Generation of a rectangular stimulation waveform arises from a rectangular command signal that opens a FET to current. (C) Triangular stimulus waveform. A centered-triangular stimulation waveform can be generated from a rectangular command signal that charges a capacitor of an integrator circuit (upward ramp), followed by a symmetric, negative command that discharges the capacitor (downward ramp).

Ten adult Sprague-Dawley rats were anesthetized with intraperitoneal injections of Nembutal (pentobarbital sodium). The left hind leg was shaved and an incision was made along the posterior aspect of the hind leg and thigh. A 5–10 mm length of the sciatic nerve was exposed under microscopic dissection proximally from the popliteal fossa. The gastrocnemius-soleus muscle complex was dissected, and the calcaneal (Achilles) tendon was severed from its distal attachment at the heel. The ipsilateral tibia was stabilized to the experimental rig via a clamp, and the calcaneal tendon was tethered to a force transducer with 1–2 N of passive tension. A monopolar nerve cuff electrode was placed on the exposed portion of the sciatic nerve as shown in Figure 2a. The electrode had a J-shaped cross-section and was made of silastic rubber with a 3 mm×1 mm rectangular platinum contact for current delivery (the 1 mm dimension was along the longitudinal axis of the nerve) [9]. A button-return electrode (Ag/AgCl) was placed subcutaneously on the dorsum of the rat and sutured into place.

Stimulation was performed by current injection into the sciatic nerve via the monopolar nerve cuff electrode. The force transducer quantified the force generated in the gastrocnemius-soleus-muscle complex. Threshold was estimated by delivering a 1 Hz train of stimulation pulses to the nerve, each with fractionally higher current amplitude than the previous pulse (incremental resolution was typically a few percent of threshold). As the current amplitude increased above threshold, the force level plateaued at the maximal contractile force (MCF). The MCF activation threshold amplitude was then back calculated from the first pulse that resulted in the MCF.

### Stimulation Waveform Comparison

The effect of waveform shape on the energy required to activate neural tissue was studied in eight Sprague Dawley rats. Stimulation was performed with either biphasic centered-triangular or rectangular waveforms [7] (Figure 2bc). Waveforms were designed with an interphase interval of 100 µs to ensure efficient biphasic stimulation (van den Honert and Mortimer 1979, McKay and Henshall 2003). Figure 2 shows the waveforms with simplified schematics representing how they could be generated by an IPG. For each animal, the biphasic stimulus waveforms were delivered to the sciatic nerve using a computer controlled function generator (AFG3021B, Tektronix, Inc., Beaverton, OR, USA) coupled to a current-controlled output stage (bp Isolator, FHC Inc., Bowdoin, ME, USA). The order in which the two waveform shapes (triangular or rectangular) were evaluated was randomized into four statistical blocks (two repeats of each waveform type). Within each block, six PWs (10, 20, 50, 100, 200, 500 µs) were evaluated in a randomized order for the given waveform. The activation threshold was determined for each trial (*i.e.* each waveform and pulse combination within a block). Statistical differences were calculated using a paired t-test at each PW using the Matched Pairs tool in statistical software JMP (Version 9, SAS Institute Inc., Cary, NC, 1989–2010). Threshold p-value for statistical significance (Probability<|t|) was set at 0.05.

### Stimulation Waveform Energy

The pulse energy required to excite a neural population is described by the following equation:
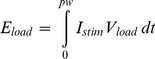
(1)where *E_load_* is the energy dissipated in the load (electrode+tissue), *I_stim_* is the peak current pulse amplitude, *V_load_* is the load voltage, and PW is the pulse-width. These values can then be used to calculate the charge (Q) for a given stimulus pulse:
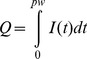
(2)where I(t) is the instantaneous current. Charge and charge density are recognized as the important values in calculating the possible risk for stimulation induced tissue damage [10].

The stimulator load consisted of the stimulating/return electrodes and the tissue, and is a significant dissipater of stimulator energy. The load voltage can be approximated by a linear function of the load impedance. Typical clinical neurostimulation devices are programmed with rectangular pulses with relatively short PWs (60–90 µs). However, both theoretical [5] and experimental [11] data suggest that the energy dissipated in a tissue load at the activation threshold exhibits a non-monotonic, U-shaped relationship with PW, reaching a minimum near 200 µs. In addition, non-rectangular stimulus pulses (e.g. Gaussian or centered-triangular) have the potential to further reduce the energy consumption necessary for neural activation [6], [7].

During stimulation at the threshold amplitude, the signals *V_load_*, and *I_stim_* (measured across a series 1 kΩ resistor) were recorded by an Agilent DSO6014A data-logging oscilloscope (Agilent Technologies, Inc., Santa Clara, CA, USA) over the duration of the stimulation pulse. *I_stim_* and *V_load_* signals were acquired using differential scope probes to prevent incidental grounding of circuit nodes with a probe. Sampling rate was adjusted to ensure acquisition of at least 500 samples over the pulse duration. Energy consumption was then calculated for each pulse using Equation 1.

### Adjustable Compliance Voltage

**Figure 3:**
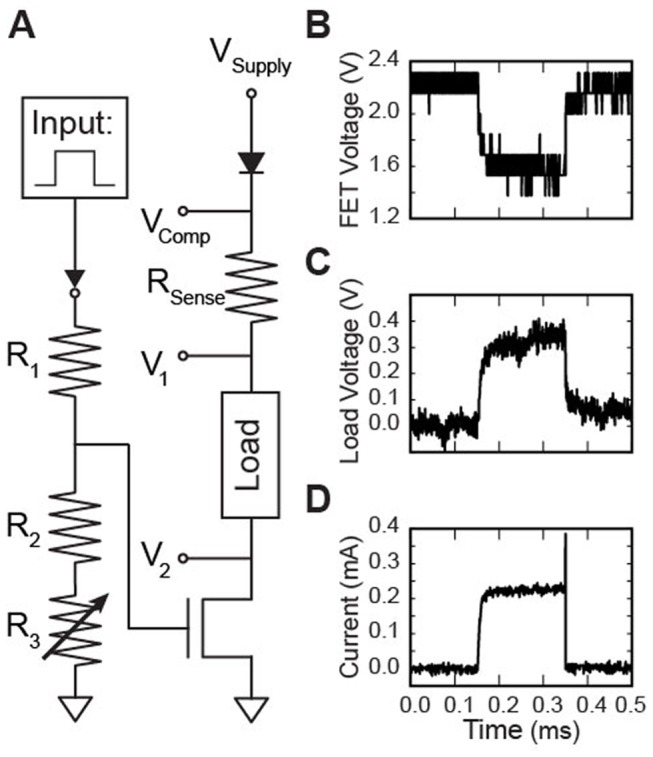
Adjustable compliance stimulator. (A) Experimental stimulator design. (B–D) Voltage/Current traces during a single trial of stimulation with a 200 µs rectangular waveform at threshold. Stimulation with the minimum calculated compliance voltage (2.8 V). (B) Voltage across the FET. (C) Voltage across the tissue and electrode load. (D) Stimulation current.

The effect of compliance voltage on the amount of energy consumed by the stimulator was evaluated in the same rat sciatic nerve preparation described above in ten Sprague-Dawley rats (which included the eight animals from the waveform comparison experiment). Rectangular stimulation pulses were delivered using a custom stimulator designed with an adjustable compliance voltage. A schematic of the constant-current stimulator is shown in Figure 3a. The transistor used was an N-channel field effect transistor (FET) model FDV301N (Fairchild Semiconductor, South Portland, ME, USA), that was biased to operate in the 0–2.0 mA range using our resistor network (R_1_ = 400 kΩ; R_2_ = 60 kΩ; R_3_ = 0–50 kΩ). This stimulator included flexibility in PW, pulse amplitude and compliance voltage. The PW of the stimulation current was equal to that of a 5 V trigger pulse delivered to *V_in_*. The amplitude of the current pulse was modulated by two 25 kΩ potentiometers in series (R_3_), which modulated the FET gate voltage during an active trigger pulse. The compliance voltage of this stimulator was adjusted manually using a bench power supply.

The compliance voltage was adjusted to maintain the FET in its operating range. When the trigger pulse was active, stimulation current flowed from the supply, through the load and the FET to ground (Figure 3ab). The voltage drop across the load (electrode and tissue) resulted in a drop in the voltage that was maintained across the FET when the stimulator was active. For insufficient compliance voltages, the voltage drop across the load was large enough to drop the FET out of the saturation-operating region, and the programmed current was not achieved. The programmed current was only achieved when the drain-source voltage of the FET was sufficient to keep it in the saturation region. The minimum required drain-source voltage to maintain operation is dependent on the current flowing through a given FET. In our experiments, the drain-source voltage was typically in the range 1.0–2.0 V.

For each animal, a randomized factorial experiment design was implemented to investigate the effect of compliance voltage and PW on energy consumption, measured across each of the two circuit elements (load and current source). Energy consumption was determined for each combination of three PWs (100, 200, 500 µs) and four compliance voltages. Three of the compliance voltages were fixed (5, 10 and 20 V), while the last was the minimal compliance voltage that maintained the constant current pulse shape (*V_min_*), determined iteratively by manual adjustment of the bench-top power supply. The order in which each of the four compliance voltages were evaluated was block randomized within each PW. The order in which the PWs were evaluated was randomized for each animal.

### Stimulation Energy with Adjustable Compliance

The following protocol was used to evaluate the energy consumption for each PW and compliance voltage combination. First the PW of a TTL trigger (5 V; 1 Hz) and the compliance voltage of the stimulator were programmed. R_3_ was adjusted such that the current amplitude was set to the activation threshold amplitude. *V_min_* was determined by (1) determining the threshold amplitude with a large compliance voltage, (2) reducing the compliance voltage until the stimulator could no longer generate the MCF, with associated distortion of the constant-current stimulation pulse shape. Once the threshold amplitude was found, the signals *V_comp_*, *V_FET_*, *V_load_* and *I_stim_* were acquired using the data-logging oscilloscope over the duration of the stimulation pulse (see Figure 3bcd). Briefly, *V_FET_* is the voltage drop across the FET (*V_2_*–*V_gnd_*), *V_load_* is the voltage drop across the electrode-tissue load (*V_1_*–*V_2_*) and *I_stim_* is the injected current into the electrode-tissue load, which is estimated by the voltage drop across a 1 kΩ series resistor:
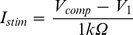
(3)


Sampling rate was adjusted to ensure acquisition of at least 500 samples over the pulse duration. *I_stim_* and *V_load_* signals were acquired using differential scope probes to prevent grounding of intermediate circuit nodes. The stimulus waveform at threshold was compared across compliance voltages to assert that the charge injection and peak current were similar for a given PW. For each pulse, total energy consumption was calculated by Equation 1. The stimulator energy was calculated as the sum of the energy consumed by the FET and by the load, since these are the values most relevant in minimizing energy consumption in IPGs.

### Model Analysis

The diameter of axons typically targeted by clinical neurostimulation applications varies substantially. For example, the typical axon diameter is only ∼2 µm for DBS applications [12], while the diameter range is more like 10–15 µm for SCS [13]. For comparison with our experimental results, the motor fiber diameter histogram peaks around 7 µm and ranges from 2–10 µm in rat peripheral nerve [14].

We performed computer simulations to investigate the relationship between axon diameter and energy consumption, across a range of PWs. Our simulations relied on the MRG mammalian myelinated axon model [15] specified at a range of different fiber diameters (2, 5.7, 7.3, 8.7, 10, 11.5, 12.8, 14, 15 and 16 µm). Simulations were performed in NEURON v7.2 [16].

Following previously described methods [7], we evaluated the theoretical energy requirements to activate populations of axons with a point source electrode. We used 10 populations of 100 parallel fibers in a bundle. The center of the bundle was located 1 mm from a point source electrode. The center nodes of each axon in each bundle were distributed 1 mm longitudinally, and 0.75 mm radially, from the point source. Stimulation was implemented with rectangular waveforms and PWs that ranged from 10–1000 µs (10 µs resolution). The stimulus current threshold for action potential initiation was determined using a binary search algorithm with accuracy <1%. Energy content was calculated from integration of the square of the stimulus current (*I_stim_*) multiplied by a fixed tissue resistance (*R_tis_ = *1.5 kΩ).
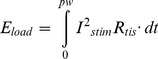
(4)


The threshold energy to recruit 50% of the fibers in each bundle was determined for each PW. The resulting energy curve for each fiber diameter was normalized to the minimum energy value for that fiber diameter. This normalization allowed for direct comparison of the energy threshold relationship as a function of PW for large and small diameter fibers (Figure 6b).

## Results

### Waveform Comparison

**Figure 4:**
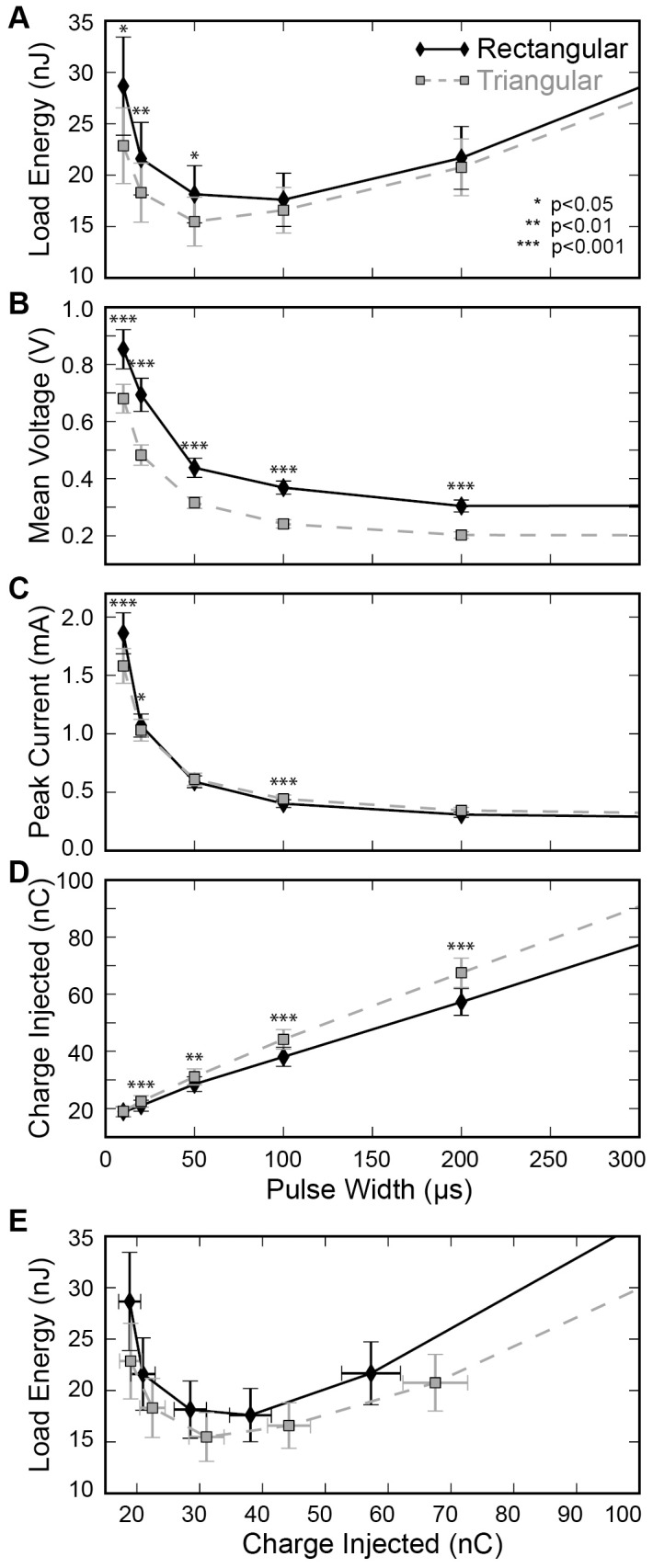
Stimulus waveform comparison. Stimulus amplitude for both the rectangular and triangular waveforms was set to the threshold amplitude for maximal contractile force in the gastrocnemius muscle. (A) The threshold energy dissipated across the combined electrode-tissue load was determined for both waveforms, with pulse-widths ranging from 10 to 500 µs. (B) The mean voltage during stimulation for both waveforms. (C) The peak current during stimulation. (D) Charge injected across the load. (E) Direct comparison of charge injection and energy dissipation. Error bars represent standard error. Significance indicated with * for p<0.05, ** for p<0.01, and *** for p<0.001.

The energy required to maximally activate the motor pool exhibited a U-shaped, non-monotonic relationship with PW for both the rectangular and triangular stimulation waveforms (Figure 4a). Energy consumption for sciatic nerve stimulation was minimized for PWs in the range of 50–100 µs. In this range, the minimum compliance voltage frequently exceeded 10 V. At all PWs tested, the energy required to generate the maximal contractile force (MCF) was greater for rectangular waveforms than for pulse-width matched centered-triangular waveforms (e.g. a 15% difference at PW = 50 µs). This difference was found to be statistically significant using a paired Student’s t-test, with p-values of 0.013, 0.0027 and 0.016 for PWs of 10, 20 and 50 µs, respectively (differences were normally distributed). When considering both waveform shapes and all PWs, the most energy efficient stimulation waveform tested was the centered-triangular waveform with a PW of 50 µs, with a mean energy requirement of 15.5 nJ (SD 9.8). This represents a 12% energy savings compared to the optimal rectangular waveform (p-value 0.006), which had a PW of 100 µs and a mean energy of 17.6 nJ (SD 10.7).

The mean voltage during stimulation was significantly higher for the rectangular waveform compared to the triangular waveform, which was the primary contributor to the energy savings seen during stimulation (Figure 4b). For a given PW, the peak current may be larger or smaller for stimulation with either waveform. However, it is important to note that the definition of PW for triangular waveforms is arbitrary [7], and it is difficult to pull significant information from a comparison based on PW alone (Figure 4c). When comparing the charge injection for each waveform (Figure 4d), stimulation with triangular waveforms injects more charge at similar PWs (p<0.01 for PW ≥20 µs). However, a comparison of charge and energy (Figure 4e), demonstrates that the triangular waveforms consume less energy for a given level of injected charge than rectangular waveforms.

### Compliance Voltage Comparison

**Figure 5:**
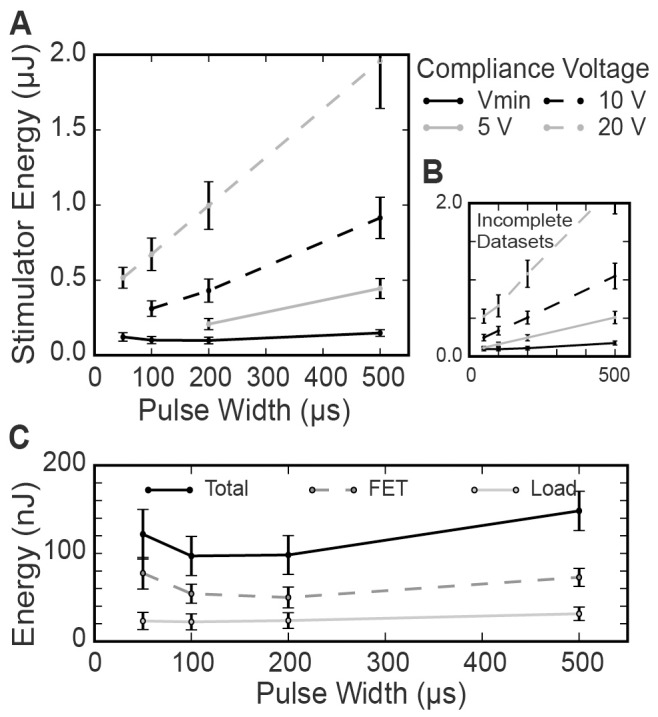
Fixed versus adjustable compliance voltage. (A) Stimulator energy consumed to maximally activate a rat sciatic nerve. Stimulator set to a compliance voltage of 5 V, 10 V, 20 V and the minimum compliance voltage. Stimulation tested across a range of pulse-widths. Incomplete datasets (compliance voltage was insufficient to activate nerve in some animals) were not included in statistical analyses. (B) For reference only, the stimulator energy consumed to activate rat sciatic nerve, with incomplete datasets included. (C) Energy consumed by the FET and by the load across a range of pulse-widths, with compliance voltage minimized. Error bars represent standard error.

The use of an adjustable compliance voltage directly affected the stimulator energy consumption (Figure 5a). For a 200 µs PW, the minimum compliance voltage achieved energy savings of 90% compared to a fixed compliance of 20 V (996 nJ vs. 98 µJ), 77% compared to 10 V (430 nJ), and even 53% compared to 5 V (207 nJ). This difference in energy consumption between the minimum compliance scenario and the fixed compliance scenarios resulted from increased energy dissipation in the current source (load energy consumption remained approximately constant for each of the compliance voltages). The difference in energy consumption was greatest for long PWs (Figure 5a). The lower fixed compliance voltages (5 V and 10 V) were not always sufficient to drive the necessary stimulation current, particularly for shorter PWs (<200 µs). Mean values were not included in Figure 5a for those compliance voltage and PW combinations in which the compliance was not sufficient for all of the animals tested (means with missing data were biased; total dataset shown in 5b for reference only). Figure 5c shows the energy consumption for the load and FET when the minimum compliance voltage was used. The energy consumption of both the load and the FET exhibited a non-monotonic relationship with PW. FET energy consumption was minimized for PWs of 100–200 µs. FET consumption was higher than load consumption in this plot as a result of the relatively low thresholds required for activation of the small rat sciatic nerve.

**Figure 6:**
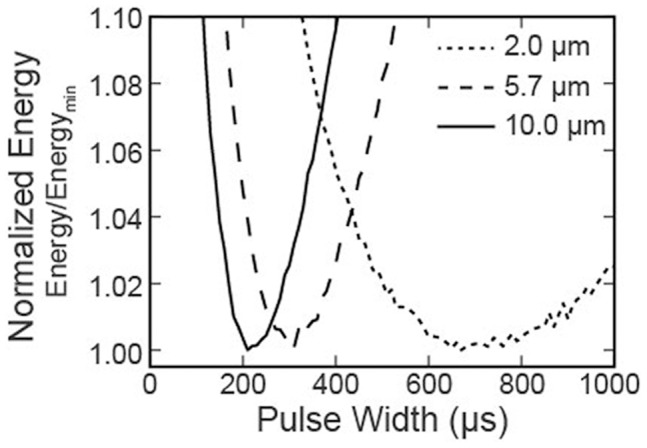
Simulated nerve bundle. Normalized energy required to recruit 50% of the axons of different diameters in a simulated nerve bundle, across a range of pulse-widths (10–1000 µs; 10 µs resolution).

### Simulation Results

Our simulations with rectangular pulses demonstrated an axon diameter dependent shift in the most energy efficient PW (Figure 6). As the axon diameter increased the optimal PW decreased. This relationship was consistent for all fiber diameters investigated (2–16 µm). In simulation, the stimulus energy required to recruit 50% of the large diameter fibers (10.0 µm) was 3.0 nJ with their most energy-efficient PW (210 µs). The 50% recruitment energy for small diameter fibers (2.0 µm) was substantially higher (54 nJ), and was found at a longer PW (670 µs).

## Discussion

The goal of this study was to investigate novel techniques to increase the energy efficiency of neural stimulation by coupling knowledge of neural membrane biophysics with electronic circuitry design. We found that the standard practice of current-controlled stimulation, which relies on a fixed compliance voltage, results in substantial energy loss compared to implementing an adjustable compliance voltage matched to the real-time needs of a specific stimulation application. In addition, we discovered alternative energy savings through the use of a centered-triangular stimulus waveform. The triangular waveform required significantly less energy to activate axons than stimulation with a conventional rectangular pulse. We also demonstrated the theoretically important role of axon fiber diameter in determining the optimal stimulation PW for a given neurostimulation application. Therefore, the combination of (1) an adjustable compliance stimulator, (2) a centered-triangular stimulus waveform and (3) an axon diameter appropriate stimulation PW, has great potential to reduce energy consumption compared to the traditional IPGs used in clinical practice.

Our data demonstrate significant power savings with triangular stimulation waveforms as compared to rectangular waveforms. Triangular waveforms achieved a 12% reduction in energy consumption when compared with the most energy efficient rectangular waveforms (optimal pulse-width and optimal compliance voltage). This experimental finding is consistent with our results from a recent simulation study that estimated the savings of 10% when comparing the energy-optimal centered-triangular waveform with the energy-optimal rectangular waveform [7]. This reduction in power consumption via non-rectangular stimulus waveforms could have important implications on the circuit design of future IPG output stages.

Over recent years, several patents have surfaced regarding implementation of variable compliance in neurostimulation applications [17], [18]. In turn, commercial neurostimulators may have already implemented variable compliance concepts into their devices. However, the implementation details, rationale, and estimated energy savings are not available in the public domain. In this paper, we demonstrate that minimization of compliance voltage dramatically improves the energy efficiency of stimulation, compared to the traditional use of a fixed compliance.

For a fixed compliance voltage, energy consumption increases monotonically with PW. This relationship arises from the monotonic increase in charge consumption with increasing PW, meaning larger energy consumption (*E = Q·V_comp_* for constant voltage). However, minimization of compliance voltage results in a U-shaped, non-monotonic relationship between pulse-width and energy consumption. This relationship arises from the asymptotic nature of the strength-duration relationship [19]. Shorter PWs require exponentially larger amplitudes, leading to exponentially increasing power (*P = I_stim_·V_comp_*). Increasing the PW reduces the current necessary for activation, so if *V_comp_* is likewise reduced, the result is a reduction in power consumption. As the stimulus PW continues to increase, the threshold stimulus asymptotically approaches the rheobase current, meaning the power also levels off. However, due to the increased duration of power consumption to maintain the pulse, stimulator energy consumption increases in a near linear fashion (Equation 1). Between these two extremes, there exists an energy-optimal PW for stimulation. Our results from stimulating large diameter axons in rat sciatic nerve suggest an energy-optimal PW of ∼100 µs.

Our simulation results show that the relationship between energy consumption and PW may also be a function of the axon diameter. The simulations predicted that large diameter axons (>10 µm), like those seen in SCS and peripheral motor stimulation, have their most efficient PW at ∼200 µs. This prediction is longer than our experimental results (∼100 µs) and our theoretical recruitment estimates (∼3 nJ) were smaller than found experimentally (∼15 nJ). These discrepancies likely arise from the simplistic nature of the electrode-tissue interface model and volume conductor electric field model used in our computer simulation.

In spite of the model limitations, the simulations do provide some insight into application of energy optimal PWs to other stimulation applications, such as DBS. Compared to peripheral nerve stimulation, stimulation targets in the central nervous system typically have axons with much smaller diameters. Based on our models, we predict that DBS would be most efficient at longer PWs [7]. This is in contrast to the current paradigm for DBS, where shorter PWs are favored (60–90 µs) to minimize charge injection by voltage-controlled stimulators, and therefore energy consumption. However, if variable compliance current-controlled stimulators were available, longer PWs would decrease the voltage requirement of stimulation, with a net reduction in energy consumption. For example, we predict potential energy savings of ∼63%, from 132 nJ (rectangular, 60 µs) to 48 nJ (centered-triangle, 550 µs), with the combination of variable-compliance and an energy-optimal stimulus waveform to activate 50% of the small diameter fibers in our simple model (Figure 6). However, energy savings with energy-optimal PWs can only be achieved by adjusting the compliance voltage to real-time requirements.

Energy consumption by the stimulator circuit is affected by the dynamics of the transistor and the capacitance of the electrode-tissue interface. The minimum drain-source voltage required to maintain operation of the FET in the saturation region increased with the current regulated by the FET (Figure 3a). This would also be true with a bipolar junction transistor; increasing the required drain-source voltage results in power loss. This type of energy loss could potentially be mitigated by feedback control of the gate voltage and optimization of the FET dynamics. Energy consumption was further impacted by the capacitance of the electrode-tissue interface. As current was delivered by the partially capacitive stimulating electrode, voltage built up across the electrode-tissue interface which generated a nearly linear rise in *V_load_* during constant current delivery (e.g. Figure 3c). This resulted in increased energy consumption, especially at long PWs, compared to an ideal, purely resistive impedance (as calculated using *I_stim_^2^*). Platinum electrodes were used in this experiment, a material that is commonly used for clinical applications such as DBS and SCS. However, materials that are less polarizable (e.g. iridium-oxide) would exhibit smaller voltage build-up during stimulation, augmenting the potential energy savings of stimulation with long PWs and an adjustable compliance voltage.

It will be important to ensure that stimulation is being delivered safely to the target tissue since use of a more efficient PW and waveform may result in more charge injected than a standard rectangular waveform and short PW (Figure 4a,d,e). The safety of a given stimulation therapy is ultimately demonstrated by application-specific chronic animal testing, however, general guidelines for charge injection limits have been established [10], [20]. The safe charge density limit will also depend on the material of the electrode. For example, most commercial stimulators use platinum/iridium electrodes and limit charge density to 50 µC/cm^2^. Alternative electrode materials such as iridium-oxide or titanium-nitride have also been used commercially, and may provide for higher charge density limits [21]. Most commercial SCS and DBS electrodes are platinum/iridium and have a surface area of 6–8 mm^2^, resulting in a charge injection limit of 3–4 µC. These limits will need to be respected in the development of a neurostimulator that uses the techniques described in this manuscript.

The findings from this study are most relevant for standard commercial electrode technologies in which a significant fraction of the electrode voltage drop occurs across the tissue. It is likely that less energy savings would be seen for very small electrodes (e.g. microelectrodes) since substantial voltage is dropped across the high electrode interface impedance (low capacitance). However, this limitation could be mitigated in part by using high performance electrode materials, as described above.

This study demonstrates that coupling circuit design with understanding of neurostimulation biophysics has potential to enable significant improvements in electrical stimulation energy efficiency. Our results may be useful in helping to design future IPGs. We predict that a combination of centered-triangular waveform shape, axon diameter optimized pulse-width, and variable compliance current-controlled pulse generators will result in measureable improvement in neuromodulation IPG battery life.
